# The Impact of COVID-19 on Cholesteatoma Diagnosis and Treatment

**DOI:** 10.7759/cureus.73306

**Published:** 2024-11-08

**Authors:** Courtney B Shires, Jeremy Yang, Mark Landry, Steven Conrad, Karuna Dewan

**Affiliations:** 1 Head and Neck Surgery, West Cancer Center, Germantown, USA; 2 Pediatric Otolaryngology, Rady Children's Hospital, San Diego, USA; 3 Otolaryngology - Head and Neck Surgery, Louisiana State University Shreveport, Shreveport, USA; 4 Pulmonary and Critical Care Medicine, Louisiana State University (LSU) Health Shreveport, Shreveport, USA; 5 Otolaryngology - Head and Neck Surgery, Louisiana State University (LSU) Health Shreveport, Shreveport, USA

**Keywords:** cholesteatoma, covid-19, delay, otology, pandemic, severity

## Abstract

Objectives: This investigation aimed to examine the effects of the COVID-19 pandemic on treatment delays and the severity of initial disease presentation in cholesteatoma patients treated in the prepandemic and pandemic periods.

Methods: This retrospective cohort study was of patients who underwent primary surgical management of cholesteatoma between October 2018 and December 2021, split between the prepandemic (October 2018 to February 2020) and pandemic (April 2020 to December 2021) time periods. Data collected included time from referral to otology clinic visit, time of diagnosis, and time of surgical interventions. The extent of cholesteatoma disease and surgery, hearing loss levels, and the need for additional surgical intervention were also considered. The datasets were compared using the Wilcoxon rank-sum test.

Results: Eighty-six patients met inclusion criteria, 36 of whom were treated prepandemic and 50 after March 2020. Of the examined variables, only time from diagnosis to surgery and case duration were significantly different between the two cohorts, with the pandemic cohort experiencing less time between initial diagnosis and surgery (51.4 days prepandemic vs. 38.4 days pandemic, p = 0.02) and shorter case duration (221.0 minutes prepandemic vs. 171.8 minutes pandemic, p = 0.0008). There was no difference between the severity of presentation in the prepandemic and pandemic populations.

Conclusions: There was no significant difference in disease severity or delays in treatment when comparing the prepandemic to the pandemic population. There was a quicker time from referral to surgery and decreased surgical times during the pandemic.

## Introduction

With significant uncertainty surrounding viral exposure and treatment during the advent of the COVID-19 pandemic, there were many delays in medical treatment for acute and chronic non-COVID-19 conditions. Patients' hesitation to leave their homes and staff shortages aiming to limit employee exposure could contribute to slower than normal management. Now that more time since the lockdowns have transpired, multiple studies in many different specialties are solidifying this link between COVID-19 and treatment delays.

Some of the more drastic changes were seen in cases of acute and life-threatening events. Ali et al. demonstrated a longer time from door to treatment in ST-segment elevated myocardial infarctions when comparing pre- and postpandemic populations, with treatment times improving after lockdown but not yet reverting to baseline [1]. A separate study out of a more resource-poor location in Latin America showed a similar significantly higher door-to-arterial puncture time during COVID-19 in patients requiring mechanical thrombectomy in stroke treatment [2]. While the authors of this second study hypothesized that the lack of resources was a contributory factor, a similar trend was seen in a diverse group of health systems.

These more acute delays could be related to issues with newly implemented isolation and safety protocols. However, similar issues in access to care were also seen with less immediately urgent medical issues. Multiple studies have shown a decrease in the number of cancer diagnoses over this time period, likely due to patients' hesitance in navigating a newly changed and unfamiliar health environment [3-5]. One investigation out of Japan helped demonstrate a brief trend of decreasing surgeries with an increase in neoadjuvant chemotherapy in the treatment of rectal cancer during the pandemic [6]. At the same time, another based out of Brazil outlined the deterioration of migraine control, a more chronic issue, that seemed directly correlated with delays in therapeutic botulinum toxin administration [7].

This study aimed to investigate diagnostic and treatment delays in the field of otolaryngology, specifically concerning cholesteatomas. Cholesteatomas are ear lesions where keratinizing stratified squamous epithelium forms a mass that can destroy local tissue. They can originate as a congenital or an acquired lesion. Acquired cholesteatomas are thought to be secondary to either chronic retraction, leading to invagination of the lateral squamous layer of the tympanic membrane, or from internal migration of this same layer after iatrogenic or other tympanic membrane disruption. This local tissue destruction can lead to secondary infection and bony erosion that can extend to the inner ear or intracranially. Cholesteatomas can present insidiously, with mild symptoms initially and often unnoticed until an infection or separate complication develops. Otorrhea and progressive hearing loss are the most commonly noted symptoms [8].

With the delays in access noted during the pandemic shutdowns and a nebulous presentation style, the possibility for more advanced initial presentations is evident. A prior study based in the UK health system concluded that longer wait times for cholesteatoma surgery were not associated with increased rates of recidivism or complications [8]. This investigation aimed to see if the pandemic resulted in delays in treatment and worsened disease severity at initial presentation for patients undergoing initial surgical intervention for cholesteatomas during prepandemic and pandemic time periods.

## Materials and methods

This study is a retrospective chart review of patients treated for cholesteatoma at a single tertiary academic center in the southeast part of the country from October 2018 to December 2021. This study was approved by the Louisiana State University Institutional Review Board Protocol 2055 on April 27, 2022. Informed consent for chart review was obtained from all patients.

Information was collected through a chart review of the electronic medical records. This was then split into two separate cohorts: prepandemic from October 2018 to February 2020 and pandemic from March 2020 to December 2021. We tried to include an equal amount of time before and after the pandemic began. Patients included were those with a diagnosed cholesteatoma and who received their first cholesteatoma surgery during the involved time period. They all had audiograms at our institution. Patients of all ages were included. All patients diagnosed with cholesteatoma were included. Patients who did not have surgery or had surgery at another institution were excluded. Patients without discoverable hearing tests were excluded. Prisoners, patients whose initial surgery was not in the appropriate time frame, and canal cholesteatomas were excluded. Patients were treated by three different surgeons. Datasets were compared using the Wilcoxon rank-sum test.

Information collected was divided into two categories: delay in treatment or disease severity. Delays in treatment criteria include the time from referral to initial encounter with an otologist, the time from initial otology encounter to surgery, and the time from referral to surgery. Disease severity criteria include a symptom score, degree of hearing loss, extent of surgery, case duration, number of subsites affected, number of ossicles involved, and the need for a secondary surgery. A symptom score was used to evaluate for severe disease. The score is comprised of a scale from 0 to 2, with 1 point for otorrhea and 1 point for any of the following: perilymphatic fistula, abscess, or facial palsy. Over the past few decades, multiple studies have proposed classification systems of cholesteatoma based on cholesteatoma extension, localization or origin, pathophysiology, and more; however, no consensus has been reached [9]. We chose to use one of these systems, understanding that combining ordinal data into a semiquantitative score causes challenges in statistical analysis.

The extent of surgery was recorded as transcanal, canal wall-up tympanomastoidectomy, or canal wall-down tympanomastoidectomy. A score of 1-3, with 1 being transcanal, 2 being canal wall-up, and 3 being canal wall-down, was used. Six total subsites were recorded for total involvement on a scale of 0-6, including the epitympanum, mesotympanum, hypotympanum, protympanum, retrotympanum, and mastoid. Ossicular involvement was measured from 0 to 3, taking note of involvement of the malleus, incus, or stapes.

## Results

A total of 86 patients were included, with 36 patients in the prepandemic and 50 patients in the pandemic period. Fifty-seven percent were male, and 43% were female. Nineteen (21.8%) were pediatric patients. Not all patients included had every variable recorded. The variables in the delay in treatment category were measured in days. The time from referral to initial encounter with an otologist was 39.6 days prepandemic in 27 patients and 36.3 days during the pandemic in 47 patients, for a difference of 3.3 days (p = 0.52). The time from diagnosis to surgery was 51.4 days prepandemic in 36 patients and 38.4 days during the pandemic in 50 patients, for a statistically significant difference of 13 days (p = 0.02). The time from referral to surgery was 41.9 days prepandemic in 27 patients and 35.0 days during the pandemic, for a difference of 6.9 days (p = 0.18).

The severity of the presentation was evaluated using the symptoms score as detailed in the methods. Among the prepandemic group, one patient was found to have a perilymphatic fistula and one was found to have facial palsy. Among the pandemic group, there was a patient with a mastoid abscess, one with a temporal lobe abscess, one perilymphatic fistula, and two patients with facial palsy. The 36 prepandemic patients had an average score of 0.97, and the 50 patients during the pandemic had an average score of 0.92. No significant difference was noted (p = 0.56). The degree of hearing loss was measured using the preoperative air-bone gap, measured in decibels. The mean hearing loss in the 28 prepandemic patients was 43.8 dB, while the mean hearing loss in the 49 patients during the pandemic was 36.3 dB. No significant difference was noted (p = 0.15).

Concerning the extent of surgery in the 36 prepandemic cohorts, 32.4% underwent transcanal surgery, 37.8% underwent a canal wall-up tympanomastoidectomy, and 29.7% underwent a canal wall-down tympanomastoidectomy. Of the 50 patients from the pandemic cohort, 14 (28%) underwent transcanal surgery, 22 (44%) underwent canal wall-up tympanomastoidectomy, and 14 (28%) underwent canal wall-down tympanomastoidectomy. They were scored on a scale as mentioned in methods, with the least invasive scoring 1 and the most scoring 3. The prepandemic mean of 1.97 saw no statistically significant difference compared to the pandemic mean of 2.00 (p = 0.87). Our surgeons were very similar in their approaches and treatment decisions based on the severity of the disease.

The case duration was measured in minutes. The 36 prepandemic patients had an average case duration of 221.0 minutes, while the 50 pandemic-era patients saw an average duration of 171.8 minutes, for a statistically significant difference of 49.2 minutes (p = 0.0008).

For the 36 prepandemic patients, there was a mean of 2.80 subsites involved. In the pandemic cohort of 50 patients, a mean of 2.68 subsites were involved. No significant difference was found (p = 0.60). The 36-patient prepandemic cohort saw a mean of 1.53 ossicles involved, while the pandemic cohort saw a mean of 1.60 ossicles involved, noting no statistically significant difference (p = 0.83). Finally, the need for a second look surgery was recorded, noting 50% of 26 prepandemic patients and 58.3% of pandemic patients requiring a second look, for an 8.3% difference (p = 0.50) (Table 1).

**Table 1:** Delays in treatment and severity of disease in prepandemic and pandemic patients The Wilcoxon rank-sum test was used to compare groups. A p value of <0.05 was considered significant ^†^1 for otorrhea and 1 for facial palsy/perilymphatic fistula/abscess ^‡^1 transcanal, 2 canal wall-up tympanomastoidectomy, and 3 canal wall-down tympanomastoidectomy ^§^1 for each of epitympanum, mesotympanum, hypotympanum, protympanum, and retrotympanum

Variables	Prepandemic	Pandemic	p value
Delays in treatment			
Referral until initial encounter (mean, days)	39.6	36.3	0.52
Initial diagnosis until surgery (mean, days)	51.4	38.4	0.02
Referral until surgery (mean, days)	41.9	35	0.18
Severity of disease			
Symptom score (mean, 0-2)^†^	0.97	0.92	0.56
Hearing loss (mean, air bone gap, dB)	43.8	36.3	0.15
Extent of surgery (mean, 1-3)^‡^	1.97	2	0.87
Case duration (mean, minutes)	221	171.8	0.0008
Subsites involved (mean, 0-6)^§^	2.8	2.68	0.6
Ossicles involved (mean, 0-3)	1.53	1.6	0.83
Need for second surgery (%)	50	58.3	0.5

## Discussion

Delays in treatment related to the COVID-19 pandemic and its lockdowns have been documented across multiple nations, specialties, and health systems. Studies have shown a variety of ways this took effect, from the pendulum shifting from surgery toward chemotherapy in rectal cancer [6] to a deterioration of long-term migraine control when there were delays in therapeutic botulinum toxin administration [7]. Diagnosis and treatment of cholesteatoma were and remain challenging. While advanced disease can be associated with significant complications like facial palsy or a perilymphatic fistula, one single surgeon study suggested that a longer delay in treatment is not associated with increased morbidity or the need for a second procedure [8].

The aim of this study was to investigate whether the unique circumstances of the COVID-19 pandemic led to delays in diagnosing and treating cholesteatomas and whether the initial presentation was more advanced. Intuitively, given the general public's hesitation for public interaction and interaction in the healthcare setting during the pandemic, one might conclude that there would be delays in diagnosis with a potential subsequent higher stage of initial presentation.

From this study, there did not appear to be any significant delay from the initial referral to the initial patient encounter or from this referral until the initial surgery. What was seen was a shorter time (51.4 days prepandemic vs. 38.4 days pandemic, p = 0.02) from diagnosis of cholesteatoma with the otologist until the initial surgery. This finding is in congruence with a UK-based study that showed that once a patient was in the health system and referred correctly, there were no significant delays in time until operation for patients who presented with cauda equina syndrome [10].

Potential explanations could include the increased availability of operating room time given the widespread cancellation of elective cases, less busy schedules of patients during this quarantine time, and an increased willingness to undergo surgery if the recovery time is perceived to be less busy. Additionally, while there is debate if delay in treatment is associated with worse disease presentation in cholesteatoma, otorrhea and hearing loss with the increased risk for infection can be debilitating symptoms. In the results presented here, no significant difference was found in the symptom score, initial hearing loss noted, the extent of surgery required, subsites involved, number of ossicles involved, or the need for further surgery when comparing prepandemic to pandemic presentations.

The only significant difference noted was in total operating time, with the cases during the pandemic requiring significantly fewer minutes (221.0 minutes prepandemic vs. 171.8 minutes pandemic, p = 0.0008). This could go hand in hand with decreased time from diagnosis to initial surgery, assuming the overall schedules for surgeons and staff are less busy. Increased availability of facilities and fewer distractions could help with planning and execution. Perhaps less resident or fellow involvement was instrumental; however, no clear conclusions can be drawn without further study. Additionally, the severity of cases presenting at the tertiary center may be changed if other smaller sites were temporarily closed or elective cases were not completed during the pandemic.

Limitations surrounding this study include that it was performed at a single institution. Local diagnostic and surgical practice patterns could affect the result. The information was gathered from three separate surgeons who operate with a rotating group of resident physicians, which could also affect variability. Overall, apart from shortened time until surgery and shortened surgical duration, there does not appear to be any effects from the pandemic on cholesteatoma diagnosis and treatment.

We have realized several more aspects of our data that could benefit practices during future pandemics. We plan to expand our data collection and include these data from 2022, which may reflect a return to normal. This time period might include a better cohort of those who suffered a delay in treatment. In a follow-up investigation, we will also consider alternative ways of classifying the time course of these patients. In addition to stratifying results into prepandemic, pandemic, and postpandemic data, we will examine the times of various COVID-19 waves and the correlation with the subsequent institutional policy changes regarding clinic and surgery scheduling.

## Conclusions

From the available data, it does not appear that the COVID-19 pandemic lead to delays in care or increased severity of disease presentation regarding cholesteatomas. Paradoxically, it seems that there may have been improved access to care, with significantly decreased time from diagnosis until surgery and decreased operating times, though further research is necessary to draw any firm conclusions.
